# The impact of subjective socioeconomic status and threat cues on intertemporal decision-making

**DOI:** 10.3389/fpsyg.2026.1778247

**Published:** 2026-02-24

**Authors:** Zhen Zeng, Yufan Chen, Weiguo Qu

**Affiliations:** 1 Cognition and Human Behavior Key Laboratory of Hunan Province, School of Education Science, Hunan Normal University, Changsha, China; 2 School of Education Science, Huaihua University, Huaihua, Hunan, China; 3 Wulingshan K-12 Educational Research Center, Huaihua University, Huaihua, Hunan, China

**Keywords:** environmental uncertainty, intertemporal decision-making, resource scarcity, subjective socioeconomic status, threat cues

## Abstract

Intertemporal decision-making is the process by which individuals weigh the trade-offs between rewards and losses at different time points. This study investigates the influence of subjective socioeconomic status on intertemporal decision-making under varying threat cues, specifically resource scarcity and environmental uncertainty. Study 1 examines the effects of subjective socioeconomic status across conditions of resource scarcity (scarce vs. control vs. abundant) on intertemporal decisions. Study 2 explores the effects of subjective socioeconomic status under conditions of environmental uncertainty (uncertain vs. control vs. certain) on intertemporal decision-making. The results indicate that: (1) subjective socioeconomic status has a significant impact on intertemporal decision-making, with individuals of lower subjective socioeconomic status showing a greater preference for immediate rewards compared to those of higher subjective socioeconomic status; (2) threat cues also significantly affect intertemporal decision-making, as individuals in the resource scarcity and environmental uncertainty groups demonstrate a stronger preference for immediate rewards compared to those in the control group and the positive cue group; (3) resource scarcity moderates the relationship between subjective socioeconomic status and intertemporal decision-making. In the resource scarcity group, individuals with lower subjective socioeconomic status prefer immediate gratification more than their higher-status counterparts; however, no significant differences in intertemporal decision preferences are observed among individuals with differing subjective socioeconomic statuses in the control and abundant groups; (4) environmental uncertainty acts as a moderator between subjective socioeconomic status and intertemporal choices, with individuals of lower subjective socioeconomic status preferring immediate gratification more in the uncertain environment than those with higher subjective socioeconomic status; again, no significant differences are noted in the control and certain environment groups regarding intertemporal decision preferences among different socioeconomic status groups. This research expands the field of intertemporal decision-making by demonstrating how culturally embedded threat cues in China (e.g., intense social mobility competition and family-based obligations) condition the SSS–intertemporal choice link, contributing to a more objective understanding of the decision preferences of individuals with low subjective socioeconomic status.

## 1 Introduction

In daily life, individuals often find themselves in dilemmas regarding time allocation, such as whether to prioritize job searching or completing a thesis as graduation approaches, or whether to spend money on travel versus investing in skill development. This process of weighing costs and benefits at different points in time is known as intertemporal choice, where individuals make decisions that involve trade-offs between outcomes in the present and future (Malkoc and Zauberman, 2019; Namboodiri et al., 2014; Liebermann and Ungar, 2002). Intertemporal choice permeates various aspects of life, including consumer behavior, educational investments, health decisions, and career choices, significantly influencing individuals’ long-term development and well-being (Guo et al., 2024; Koivusilta et al., 2024; Peeters, 2018). Therefore, a thorough investigation into the mechanisms and influencing factors of intertemporal decision-making is crucial for understanding individual behavior and formulating effective intervention strategies. In the Chinese context, intertemporal trade-offs are frequently made under structural constraints and culturally salient goals (e.g., housing affordability, education-based mobility, and family interdependence), which may reshape how individuals evaluate delayed outcomes and respond to threat cues.

Research on intertemporal decision-making typically employs experimental methods, with laboratory studies being the predominant paradigm. Classic experimental paradigms include choice tasks, matching methods, pricing tasks, and evaluation tasks (Anderson and Stafford, 2009; Zhang et al., 2023; Konstantinidis et al., 2020; Scholten et al., 2014). Choice tasks require participants to select between immediate smaller rewards and larger future rewards, measuring individuals’ delay discounting rates by adjusting the timing and amounts of the rewards (McCormack et al., 2024; Lei et al., 2012; Patt et al., 2021). The matching method allows participants to fill in amounts themselves, making the current and future values subjectively equivalent, thus assessing individuals’ judgments of future value (Zhang et al., 2023). pricing and evaluation tasks infer choice preferences by having participants evaluate events or items at specific times (Zhou et al., 2022; Khemka, 2021).

Intertemporal decision-making is influenced by a variety of factors. In terms of decision options, the magnitude and framing of rewards can affect individuals’ preferences (Reeck et al., 2021; Zilker and Pachur, 2021; Guo et al., 2024). From the perspective of decision-makers, individual characteristics such as age, gender, personality traits, emotional states, and both subjective and objective socioeconomic status can impact intertemporal choices (Luo et al., 2024; Keidel et al., 2021; Ni and Ueichi, 2024; Gulseven and Mostert, 2019). Additionally, decision contexts, including resource scarcity, inflation, cultural background, and gain-loss situations, significantly affect individual decision-making (Huijsmans et al., 2019; Xin et al., 2024). From a theoretical perspective, Life History Theory provides an integrative framework for understanding systematic individual differences in intertemporal decision-making. According to this theory, individuals’ preferences for immediate versus delayed outcomes reflect adaptive strategies calibrated by long-term exposure to environmental conditions, particularly resource availability and predictability. Among these factors, subjective socioeconomic status (SSS)—which refers to an individual’s subjective perception of their relative position within the social hierarchy—has been identified as a key psychological factor influencing intertemporal decision-making (Gunia et al., 2009). In China, SSS is often intertwined with family-based status evaluation, “face” concerns, and strong upward-mobility norms, meaning that perceived rank may carry heightened psychosocial consequences beyond material resources. Moreover, rapid social change and intensified competition in education and labor markets can make status comparisons and future security especially salient, potentially amplifying the behavioral impact of threat cues. Numerous studies have confirmed that individuals with lower subjective socioeconomic status tend to exhibit higher delay discount rates and prefer immediate smaller rewards compared to those with higher subjective socioeconomic status (Pinger et al., 2024; Peviani et al., 2024). This association is thought to reflect that individuals with low subjective socioeconomic status may experience or expect long-term resource scarcity and future uncertainty, thereby developing adaptive decision-making patterns that prioritize immediate needs (Tunney and James, 2022). Furthermore, lower subjective socioeconomic status is often associated with reduced feelings of control and self-efficacy, which may further weaken individuals’ willingness to delay gratification in pursuit of long-term goals (Kraus et al., 2012; Ren et al., 2023). Beyond these descriptive explanations, Life History Theory provides a mechanistic account of how subjective socioeconomic status shapes intertemporal decision-making through a set of interrelated cognitive processes. Specifically, individuals with lower subjective socioeconomic status tend to assign reduced subjective value to delayed rewards, as future outcomes are perceived as less reliable and attainable given prior environmental experiences. In addition, prolonged exposure to unstable or resource-scarce environments may alter individuals’ perception of temporal delay, rendering future rewards psychologically more distant or uncertain. Chronic socioeconomic disadvantage is also frequently associated with diminished self-regulatory resources, which further limits individuals’ capacity to inhibit immediate impulses in favor of long-term benefits. Taken together, these processes constitute a coherent psychological pathway linking subjective socioeconomic status to present-oriented decision preferences. Importantly, Life History Theory does not imply that such decision strategies are expressed uniformly across contexts. Rather, their behavioral expression is contingent upon situational cues that signal environmental harshness or unpredictability. To account for this contextual dependency, the present study integrates Life History Theory with a contextual sensitivity and match framework, drawing on Differential Susceptibility Theory and the Biological Sensitivity to Context model. These perspectives emphasize that individuals differ systematically in their sensitivity to environmental cues as a function of cumulative developmental experiences. Accordingly, individuals with lower subjective socioeconomic status—who are more likely to have been chronically exposed to harsh or unpredictable environments—may develop heightened sensitivity to threat-related cues. In contrast, individuals with higher subjective socioeconomic status typically possess greater psychological buffering resources, such as stronger perceived control and future certainty, which attenuate the impact of situational threats on their decision-making processes.

According to Life History Theory, individuals chronically exposed to harsh or unpredictable environments tend to adopt a “fast” life history strategy, prioritizing immediate survival and short-term rewards. Consequently, when threat cues (such as resource scarcity or environmental uncertainty) are present, individuals with low subjective socioeconomic status exhibit a significantly reduced sense of control over the future and a diminished perceived value of future outcomes, thereby displaying a stronger preference for immediate rewards. Importantly, although resource scarcity and environmental uncertainty are both conceptualized as threat cues, they are theorized to operate through partially distinct psychological mechanisms. Resource scarcity primarily signals an immediate imbalance between needs and available resources, which induces cognitive tunneling and attentional narrowing toward urgent, short-term outcomes, thereby amplifying the perceived “needs–resources” gap. In contrast, environmental uncertainty undermines individuals’ confidence in future predictability and long-term planning efficacy, reducing the subjective value of delayed rewards even when immediate needs are not acutely threatened. Thus, while both threat cues promote present-oriented decision-making, they do so via different cognitive routes—one through immediate need amplification, and the other through future value devaluation. In contrast, individuals with high subjective socioeconomic status, having developed in relatively stable environments, are more inclined toward a “slow” life history strategy. They possess greater psychological buffering capacity and are expected to respond less intensely to threat cues; thus, their decision preferences are likely to be less influenced by such cues. Within this integrated theoretical framework, threat cues are conceptualized not merely as background stressors, but as situational activators that selectively trigger or suppress SSS-related decision tendencies depending on person–context fit. Although prior research has established the relationship between subjective socioeconomic status and intertemporal decision-making (Pinger et al., 2024; Peviani et al., 2024; Tunney and James, 2022), these studies have predominantly been conducted within Western cultural contexts. The present study aims to examine the applicability of this relationship in a Chinese cultural setting and further investigate the moderating role of threat cues. In particular, the present study distinguishes between two theoretically distinct threat cues—resource scarcity and environmental uncertainty—and examines whether they operate through different psychological pathways while interacting with subjective socioeconomic status to shape intertemporal decision-making. Based on the above reasoning, we propose the following hypothesis: H1: Subjective socioeconomic status has a significant main effect on intertemporal decision-making, with individuals of lower subjective socioeconomic status being more inclined to choose immediate rewards than those of higher subjective socioeconomic status. Although this main effect has been widely validated in Western cultural contexts, examining it within the specific socio-cultural context of China serves as a necessary empirical foundation rather than the central contribution of the present study. Establishing this baseline effect allows for a clearer interpretation of subsequent analyses that focus on boundary conditions and interaction effects.

At the same time, threat cues in the environment, particularly resource scarcity and environmental uncertainty, have also been shown to significantly modulate intertemporal decision-making behavior (Yu et al., 2024). Resource scarcity, whether it is actual material deprivation or a perceived lack thereof, can induce a “scarcity mindset” or “cognitive tunneling,” forcing individuals’ cognitive resources to focus on immediate pressing needs, thus increasing their preference for immediate rewards (Bian and Wu, 2023; Wood, 1998; Kraft et al., 2022). Environmental uncertainty, such as concerns about future economic conditions and job stability, may reduce individuals’ sense of control and perceived value regarding future outcomes, leading them to favor certain immediate rewards and exhibit higher delay discounting. These responses can also be understood as adaptive mechanisms to ensure short-term survival and benefits in potentially dangerous or unstable environments (Pepper and Nettle, 2017). Based on this, we propose Hypothesis 2: Resource scarcity has a significant main effect on intertemporal decision-making, with the resource scarcity group being more inclined to choose immediate rewards than the control group and the resource abundance group; and Hypothesis 3: Environmental uncertainty has a significant main effect on intertemporal decision-making, with the environmental uncertainty group being more inclined to choose immediate rewards compared to the control group. Importantly, these hypotheses are not intended to introduce novel main effects, but to establish the basic impact of distinct threat cues on intertemporal decision-making. By confirming these baseline effects, the present study creates a necessary reference point for examining whether and how different threat cues function as contextual activators that differentially interact with subjective socioeconomic status. Importantly, these two cues may be especially dissociable in China: resource scarcity often reflects immediate budget constraints, whereas environmental uncertainty is frequently tied to macro-level volatility and institutional factors (e.g., employment stability and long-term opportunity expectations). This contextual distinction allows a sharper test of whether “scarcity” and “uncertainty” activate different psychological routes (e.g., cognitive tunneling vs. diminished future controllability), thereby generating potentially different SSS-contingent patterns.

Furthermore, extensive research has established the impact of subjective socioeconomic status, resource scarcity, and environmental uncertainty on intertemporal decision-making (Pinger et al., 2024; Peviani et al., 2024; Tunney and James, 2022; Huijsmans et al., 2019). However, most of these studies have been conducted within Western cultural contexts. The present study aims to examine the applicability of these effects in a Chinese cultural setting, and to further investigate how different types of threat cues (resource scarcity vs. environmental uncertainty) may exert differential effects among individuals with varying levels of subjective socioeconomic status. Therefore, the primary focus of this study is not merely to replicate established findings, but to lay the groundwork for subsequent research on interactive mechanisms by exploring how these effects manifest within the Chinese cultural context. Existing research has either treated threat cues in a general and undifferentiated manner (Yang and Zhang, 2022) or has not sufficiently examined how threat cues condition the effects of subjective socioeconomic status on intertemporal decision-making. Conceptually, the present study treats threat cues (resource scarcity and environmental uncertainty) as contextual moderators, whereas subjective socioeconomic status is conceptualized as a relatively stable individual difference that determines susceptibility to these cues. To gain a deeper understanding of these differences, this study draws upon Differential Susceptibility Theory and the Biological Sensitivity to Context model. These theoretical frameworks posit that individuals differ significantly in their environmental sensitivity, particularly when confronted with stress or threat cues (Donovan et al., 2024; Li et al., 2020). Specifically, early life experiences—particularly chronic exposure to resource-scarce or unpredictable environments among individuals with low subjective socioeconomic status—may foster heightened environmental sensitivity, analogous to an “orchid” phenotype. Such individuals are more responsive to negative environmental cues, and their behavioral patterns are more readily triggered and shaped by contextual changes. In contrast, individuals with high subjective socioeconomic status may align more closely with a “dandelion” phenotype, exhibiting generally stronger adaptive capacity and more stable responses to environmental fluctuations (Boyce and Ellis, 2005).

Building on this, we propose that threat cues function not merely as external information for individuals with low subjective socioeconomic status, but rather as a trigger that activates their deep-seated sense of insecurity and adaptive decision-making patterns. According to Life History Theory, individuals chronically exposed to harsh or unpredictable environments tend to adopt a “fast” life history strategy, prioritizing immediate survival and short-term rewards. Consequently, when threat cues (such as resource scarcity or environmental uncertainty) are present, individuals with low subjective socioeconomic status exhibit a significantly reduced sense of control over the future and a diminished perceived value of future outcomes, thereby displaying a stronger preference for immediate rewards. In contrast, individuals with high subjective socioeconomic status, having developed in relatively stable environments, are more inclined toward a “slow” life history strategy. They possess greater psychological buffering capacity and are expected to respond less intensely to threat cues; thus, their decision preferences are likely to be less influenced by such cues. Therefore, this study aims to systematically examine how the interaction between subjective socioeconomic status and two core threat cues (resource scarcity and environmental uncertainty) influences intertemporal decision-making. Importantly, the present study does not conceptualize this interaction as a simple amplification of disadvantage. Instead, drawing on Life History Theory and a context-specific activation perspective, we propose that threat cues function as situational activators that selectively trigger latent decision-making strategies shaped by long-term environmental calibration. In line with this framework, the following hypotheses focus on whether threat cues moderate the association between subjective socioeconomic status and intertemporal decision-making, as reflected by statistically significant interaction effects.

The following interaction hypotheses are proposed Hypothesis 4: There is a significant interaction between subjective socioeconomic status and resource scarcity, indicating that resource scarcity moderates the association between SSS and intertemporal decision-making. Under conditions of resource scarcity, individuals with low subjective socioeconomic status will exhibit a stronger preference for immediate rewards compared to those with high subjective socioeconomic status, Crucially, this hypothesis is grounded in the expectation that resource scarcity serves as a contextual trigger that activates fast life history strategies among low-SSS individuals, rather than merely intensifying an already existing preference for immediacy. Accordingly, SSS-related differences are expected to be minimal under conditions of resource abundance, but to emerge more distinctly once scarcity cues are present. Hypothesis 5: There is a significant interaction between subjective socioeconomic status and environmental uncertainty, indicating that environmental uncertainty moderates the association between SSS and intertemporal decision-making. Under conditions of environmental uncertainty, individuals with low subjective socioeconomic status will show a greater inclination toward immediate rewards than their high subjective Socioeconomic status counterparts, Similarly, environmental uncertainty is theorized to function as an activating cue that undermines future-oriented planning primarily among individuals whose developmental experiences have calibrated them toward heightened sensitivity to instability. This implies a context-dependent expression of SSS-related decision strategies, rather than a uniform vulnerability amplification across all conditions.

To test these hypotheses, this study conducts two experiments to systematically investigate the effects of subjective socioeconomic status and threat cues on intertemporal decision-making. Experiment 1 employs a situational priming method to manipulate participants’ perceptions of resource availability (scarcity, control, abundance) and combines measurements of subjective socioeconomic status to explore the interaction of resource scarcity threats with subjective socioeconomic status in the standardized delay discounting task. Experiment 2 primes different expectations of future environments (uncertain, control, certain) and combines measurements of subjective socioeconomic status to analyze the interaction between environmental uncertainty threats and subjective socioeconomic status on intertemporal decision preferences, supplemented by exploratory measures such as reaction times or physiological indicators (e.g., skin conductance) to further reveal potential underlying mechanisms.

Taken together, the primary contribution of the present study does not lie in the replication of established main effects, but in revealing the boundary conditions under which these effects emerge. Specifically, the study focuses on the activating role of threat cues and systematically compares the differential mechanisms of resource scarcity and environmental uncertainty. By adopting this perspective, the present research moves beyond demonstrating whether subjective socioeconomic status and threat cues matter, to addressing when, how, and for whom they matter in shaping intertemporal decision-making.

## 2 Experiment 1: the impact of subjective socioeconomic status and resource scarcity on intertemporal decision-making

### 2.1 Objective and hypotheses

The aim of Experiment 1 is to explore the preferences in intertemporal decision-making among individuals with different subjective socioeconomic statuses under conditions of resource scarcity. The research hypotheses are as follows: H1: The main effect of subjective socioeconomic status is significant. Individuals with low subjective socioeconomic status will have a higher rate of choosing immediate options compared to those with high subjective socioeconomic status in intertemporal decision-making. H2: The main effect of resource scarcity is significant. The group experiencing resource scarcity will have a higher rate of choosing immediate options compared to the control group and the resource-rich group. H3: There is an interaction effect between subjective socioeconomic status and resource scarcity. In the resource-scarce group, individuals with low subjective socioeconomic status will prefer immediate options more than those with high subjective socioeconomic status in intertemporal decision-making.

### 2.2 Methods

#### 2.2.1 Participants

All procedures were conducted in accordance with established norms for behavioral experimental research, and the study design, sample size determination, and statistical analyses were specified prior to data collection. Using G*Power 3.1 software, 2 × 3 analysis of variance (ANOVA) was conducted, setting *α* = 0.05, 1-*β* = 0.8, and an effect size of 0.25, which indicated that a sample size of 158 participants was needed. Participants were selected from employees in service-oriented and technical sectors, excluding those with a psychology background. A total of 300 qualified participants were recruited, aged between 18 and 60 years (*M* ± SD = 33.41 ± 9.48 years). They were randomly assigned to one of three groups: resource scarcity, control, and resource-rich. The resource scarcity group consisted of 116 participants (high subjective socioeconomic status: *n* = 48; low subjective socioeconomic status: *n* = 64; male: *n* = 70; female: *n* = 42; *M* ± SD = 31.37 ± 9.26 years), the control group included 100 participants (high subjective socioeconomic status: *n* = 32; low subjective socioeconomic status: *n* = 68; male: *n* = 57; female: *n* = 43; *M* ± SD = 34.17 ± 8.94 years), and the resource-rich group had 88 participants (high subjective socioeconomic status: *n* = 32; low subjective socioeconomic status: *n* = 56; male: *n* = 34; female: *n* = 54; *M* ± S*D* = 35.16 ± 9.96 years). All participants were required to have basic reading and comprehension skills, and none had previously participated in similar experimental studies. The research was approved by the Ethics Committee of the Psychology Department at Hunan Normal University (Approval No: 2025-527).

Although random assignment was employed, unequal sample sizes across the three experimental conditions emerged due to participant attrition and data exclusion during the manipulation-check stage. Specifically, participants who failed the manipulation check (i.e., whose perceived resource availability ratings did not correspond to their assigned condition) or who provided incomplete responses were excluded prior to analysis. This resulted in modest discrepancies in final group sizes, a pattern commonly observed in experimental studies involving psychological priming procedures. Importantly, all final group sizes exceeded the minimum sample size required by the *a priori* power analysis.

#### 2.2.2 Experimental design

This experiment employed a 2 (subjective socioeconomic status: high vs. low) × 3 (resource scarcity: scarce vs. control vs. abundant) between-subjects design. The independent variables were the classification of subjective socioeconomic status and the level of resource scarcity. Subjective socioeconomic status (SSS) was divided into high and low groups using a median split.

#### 2.2.3 Measures

##### 2.2.3.1 Childhood subjective socioeconomic status questionnaire

This questionnaire is adapted from Griskevicius et al. (2013) and consists of three items on a seven-point Likert scale (1 = strongly disagree, 7 = strongly agree) measuring perceived family financial sufficiency, relative affluence of living area, and family wealth compared to peers during childhood (ages 6–12). A higher total score indicates higher childhood SSS (Griskevicius et al., 2013). Sample items included: “My family usually had enough money for the things we needed,” and “Compared with other families around us, my family was relatively wealthy during my childhood.” Higher scores indicated higher perceived childhood SSS. In the present sample, the scale demonstrated good internal consistency (Cronbach’s *α* = 0.82).

##### 2.2.3.2 Adolescence subjective socioeconomic status questionnaire

Adapted from the childhood SSS questionnaire (Griskevicius et al., 2013), this tool uses similarly phrased items to measure SSS during adolescence (ages 13–18) on a seven-point Likert scale. A higher total score reflects higher adolescent SSS (Griskevicius et al., 2013). The adolescent SSS scale employed items parallel in structure to the childhood SSS questionnaire, adapted to reflect the period of adolescence (ages 13–18). Sample items included: “During my teenage years, my family’s financial situation was better than that of most families around us.”The scale showed acceptable internal reliability in the current sample (Cronbach’s α = 0.83).

##### 2.2.3.3 Adult subjective socioeconomic status questionnaire

This study employs a Chinese version of Adler’s MacArthur Scale. It consists of two ladder-ranking items (1–10) assessing one’s standing relative to their province and to people around them. Higher self-ratings indicate higher perceived SSS.

Adult SSS was measured using the Chinese version of the MacArthur Scale of Subjective Social Status. Participants were asked to place themselves on a 10-rung ladder representing their perceived social standing relative to others in their province and in their immediate social environment. Higher ladder positions reflected higher perceived SSS.

##### 2.2.3.4 Selection of resource scarcity materials

Based on relevant literature (Hill et al., 2012; Mittal and Griskevicius, 2014), three types of reading materials were used: resource scarcity (e.g., economic recession), neutral control (e.g., jellyfish characteristics), and positive resource abundance (e.g., economic recovery) materials (Table 1).

**Table 1:** Results of one-way ANOVA for manipulation check of resource scarcity.

Levels of resource scarcity	*n*	*M*	SD	*F*	*p*
Resource scarcity	5	5.44	0.98	7.62	0.007
Resource abundance	5	3.08	1.11		
Resource control	5	4.16	0.74		

##### 2.2.3.5 Delayed decision-making task

To assess the effectiveness of the resource scarcity manipulation, participants completed a brief manipulation check immediately after the reading and writing tasks. The manipulation check focused on participants’ current perceived economic constraint and resource insufficiency, rather than general mood or arousal. Representative items included: “At this moment, I feel that financial resources are scarce,” “Right now, I feel that I must be very careful about spending money,” and “I feel that my current economic resources are insufficient to meet my needs.” All items were rated on a 7-point Likert scale (1 = strongly disagree, 7 = strongly agree), and scores were averaged, with higher values indicating stronger perceived economic scarcity.

The manipulation check items and descriptive statistics for each experimental condition (scarcity, control, abundance) are reported in Table 1, allowing an objective evaluation of the manipulation’s effectiveness.

**Figure 1:**
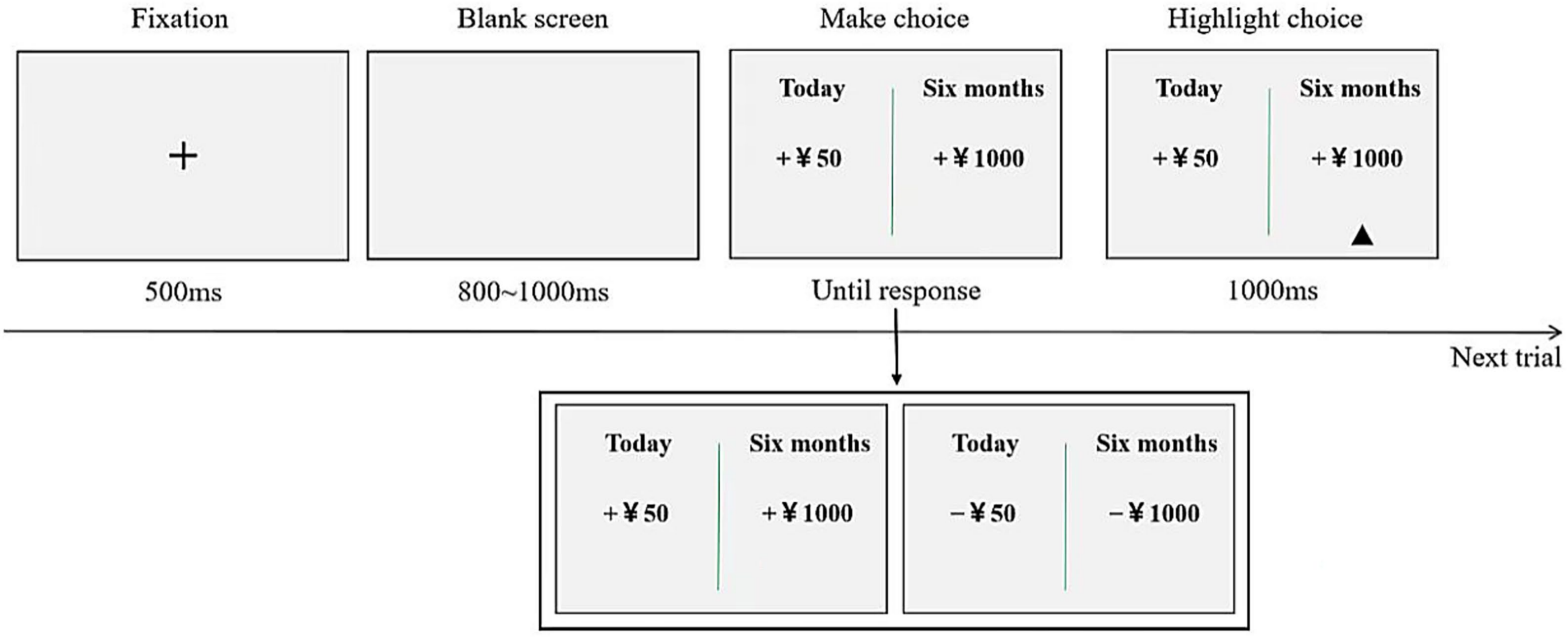
Flowchart of the intertemporal decision-making paradigm.

The task was based on the classic intertemporal choice paradigm (Griskevicius et al., 2011), consisting of a total of 19 pairs of options. The immediate option was a fixed amount of 50 yuan, while the delayed option varied between 100 and 1,000 yuan, increasing in increments of 50 yuan, with a delay of 6 months for all delayed rewards. The experimental tasks were presented using a computer (Figure 1). In each trial, a “+” symbol appeared in the center of the screen for 500 milliseconds to signal participants to prepare for the task. Following this, two options were displayed: the left option represented the immediate reward and the right option represented the delayed reward. Participants were instructed to make their choices based on their actual preferences, pressing the “F” key for the left option and the “J” key for the right option. Upon pressing either key, a small triangle appeared below the selected option for 1,000 milliseconds to confirm the choice. The task then proceeded to the next trial. The entire experiment was divided into two blocks, each containing 19 trials. The primary measure was the percentage of trials in which participants chose the immediate option.

#### 2.2.4 Experimental procedure

In this experiment, all participants completed the tasks in independent small rooms, using E-Prime 2.0 software (provided by Psychological Software Tools, Pittsburgh, Pennsylvania, USA) for stimulus presentation and behavioral data collection. The experiment consisted of five parts. First, all participants were administered questionnaires regarding their subjective socioeconomic status during childhood, adolescence, and adulthood to gather relevant background information. Subsequently, participants were randomly divided into three groups: the first group read priming materials related to resource scarcity, the second group read materials concerning daily affairs, and the third group read priming materials related to resource abundance. Following this, each group completed the corresponding writing tasks. After completing these tasks, all participants were required to fill out a resource availability questionnaire to assess the effectiveness of the priming materials. Participants then engaged in an intertemporal decision-making task to evaluate their decision-making behavior. In the data analysis, the researchers examined the consistency of subjective socioeconomic status across different life stages and selected childhood subjective socioeconomic status scores as the independent variable for subsequent analyses.

#### 2.2.5 Data analysis and statistics

E-Prime 2.0 was used for experimental programming and behavioral data collection, and SPSS 23.0 was employed to conduct Pearson correlation analysis on subjective socioeconomic status scores across childhood, adolescence, and adulthood, a one-way analysis of variance on the manipulation check scores for resource scarcity, and a two-way analysis of covariance along with simple effects analysis on the ratio of immediate choices (SS ratio) in intertemporal decision-making. Although interaction terms were tested statistically, theoretical interpretation throughout the study conceptualizes threat cues as moderators that shape the expression of SSS-related differences, rather than treating SSS itself as the contextual moderator.

### 2.3 Results

#### 2.3.1 Consistency test of subjective socioeconomic status

A Pearson correlation was conducted to analyze the consistency among subjective socioeconomic status during childhood, adolescence, and adulthood. As indicated in the table below, there was a highly significant positive correlation between childhood subjective socioeconomic status (SSS) and adolescent SSS, with *r* = 0.88, *p* < 0.001. Additionally, childhood Subjective Socioeconomic Status SSS was also significantly positively correlated with adult SSS, *r* = 0.47, *p* < 0.001. Furthermore, a significant positive correlation was found between adolescent SSS and adult SSS, *r* = 0.50, *p* < 0.001. The results demonstrate that all three stages of subjective socioeconomic status—childhood, adolescence, and adulthood—are significantly positively correlated, with correlation coefficients exceeding 0.4, indicating a moderate level of positive correlation. This suggests a strong consistency in subjective socioeconomic status across childhood, adolescence, and adulthood (Table 2).

**Table 2:** Consistency test of subjective socioeconomic status scores across different life stages.

Subjective socioeconomic status	*M*	SD	Childhood SSS	Adolescence SSS	Adulthood SSS
Childhood SSS	3.68	1.65	1		
Adolescence SSS	3.73	1.65	0.88***	1	
Adulthood SSS	5.26	1.62	0.47***	0.50***	1

***p < 0.001.

#### 2.3.2 Manipulation check for resource scarcity priming

A one-way ANOVA was conducted with resource scarcity (scarcity vs. control vs. abundance) as the independent variable and the average scores of the scarcity check as the dependent variable. The results indicate significant differences in the scarcity check scores across different levels of resource scarcity, *F*(2, 297) = 17.12, *p* < 0.001. Post-hoc comparisons reveal that the scarcity group (*M* ± SD = 5.04 ± 1.13) had significantly higher scarcity check scores than both the abundance group (*M* ± SD = 4.18 ± 1.13) and the control group (*M* ± *SD* = 4.19 ± 1.38), confirming that the manipulation check was successful (Table 3).

**Table 3:** Results of the one-way ANOVA for the manipulation check of resource scarcity priming.

Levels of resource scarcity	*n*	*M*	SD	*F*	*p*
Resource scarcity	112	5.04	1.13	17.12	<0.001
Resource abundance	88	4.18	1.13		
Resource control	100	4.19	1.38		

#### 2.3.3 Analysis of intertemporal decision-making: main and interaction effects

A two-way completely randomized ANOVA was conducted with the level of resource scarcity (Scarcity vs. Control vs. Abundance) and subjective Socioeconomic social status (Low SSS vs. High SSS) as independent variables, while controlling for gender and age. The dependent variable was the SS ratio of intertemporal decision-making (Table 4).

**Table 4:** Descriptive statistics of the SS ratio for intertemporal decision-making under conditions of resource scarcity and subjective social status.

Variables in the study	Subjective socioeconomic status	Scarcity	Abundance	Control	Total
Intertemporal decision SS ratio(*M* ± *SD*)	Low SSS	0.67 ± 0.30	0.22 ± 0.30	0.35 ± 0.32	0.42 ± 0.36
	High SSS	0.38 ± 0.37	0.17 ± 0.27	0.22 ± 0.33	0.27 ± 0.35
	Total	0.55 ± 0.36	0.20 ± 0.29	0.31 ± 0.33	0.36 ± 0.36

After controlling for the effects of gender and age, the main effect of Subjective Socioeconomic Status (SSS) was significant, *F*(1, 292) = 14.55, *p* < 0.01, *η2* = 0.05 (Table 5). The intertemporal decision SS ratios for the low SSS group (*M* ± SD = 0.42 ± 0.36) were significantly higher than those of the high SSS group (0.27 ± 0.35). The main effect of resource scarcity was also significant, *F*(2, 292) = 25.98, *p* < 0.001, *η*^2^ = 0.15. *Post hoc* multiple comparisons indicated that the SS ratios for the wealthy group (*M* ± *SD* = 0.20 ± 0.29) did not significantly differ from those of the control group (*M* ± SD = 0.31 ± 0.33), *p* > 0.05; however, both were significantly lower than the SS ratio of the scarcity group (*M* ± SD = 0.55 ± 0.36), *ps* < 0.001. There was a significant interaction between SSS and resource scarcity, *F*(2, 292) = 3.34, *p* = 0.04, *η*^2^ = 0.02, which necessitated a simple effects analysis.

**Table 5:** Interpersonal effects of subjective socioeconomic status and resource scarcity on the ss ratio in intertemporal Decision-Making.

Source of error	*SS*	*df*	*MS*	*F*	*p*	*η* ^2^
Intercept	2.15	1	2.15	20.86	0.01	0.07
Gender	0.03	1	0.03	0.30	0.58	0.00
Age	0.01	1	0.01	0.09	0.77	0.00
Resource group	5.35	2	2.68	25.98	0.01	0.15
SSS	1.50	1	1.50	14.55	0.01	0.05
Resource group * SSS	0.69	2	0.35	3.34	0.04	0.02
Error	30.09	292	0.10			
Total	79.08	300				

The results of the simple effects analysis showed that, for individuals with low subjective socioeconomic status, the SS ratios of intertemporal decisions varied significantly across different resource scarcity conditions, *F*(2,292) = 30.76, *p* < 0.001, *η*^2^ = 0.17. Further *post hoc* comparisons revealed that the scarcity group (*M* ± SD = 0.67 ± 0.30) had a significantly higher SS ratio compared to the control group (*M* ± SD = 0.35 ± 0.32), *p* < 0.001. Additionally, the SS ratio of the control group (0.35 ± 0.32) was significantly higher than that of the wealth group (*M* ± SD = 0.22 ± 0.30), *p* < 0.05. For individuals with high subjective socioeconomic status, the SS ratios also differed significantly across resource scarcity conditions, *F*(2,292) = 4.67, *p* < 0.05, *η2* = 0.03. Post hoc tests indicated that the scarcity group (*M* ± SD = 0.38 ± 0.37) had a significantly higher SS ratio than both the wealth group (*M* ± SD = 0.17 ± 0.27) and the control group (*M* ± SD = 0.22 ± 0.33), *p* < 0.05 (Table 6).

**Table 6:** Post hoc pairwise comparisons of intertemporal decision SS ratios under resource scarcity group conditions.

(I)Resource group	(J)Resource group	Mean difference	95%CI	
			LLCI	ULCI
Scarcity	Abundance	0.33*	0.23	0.42
	Control	0.24*	0.15	0.33
Abundance	Scarcity	−0.33*	−0.42	−0.23
	Control	−0.09	−0.17	0.01
Control	Scarcity	−0.24*	−0.33	−0.15
	Abundance	0.09	−0.01	0.19

*p < 0.05.

**Figure 2:**
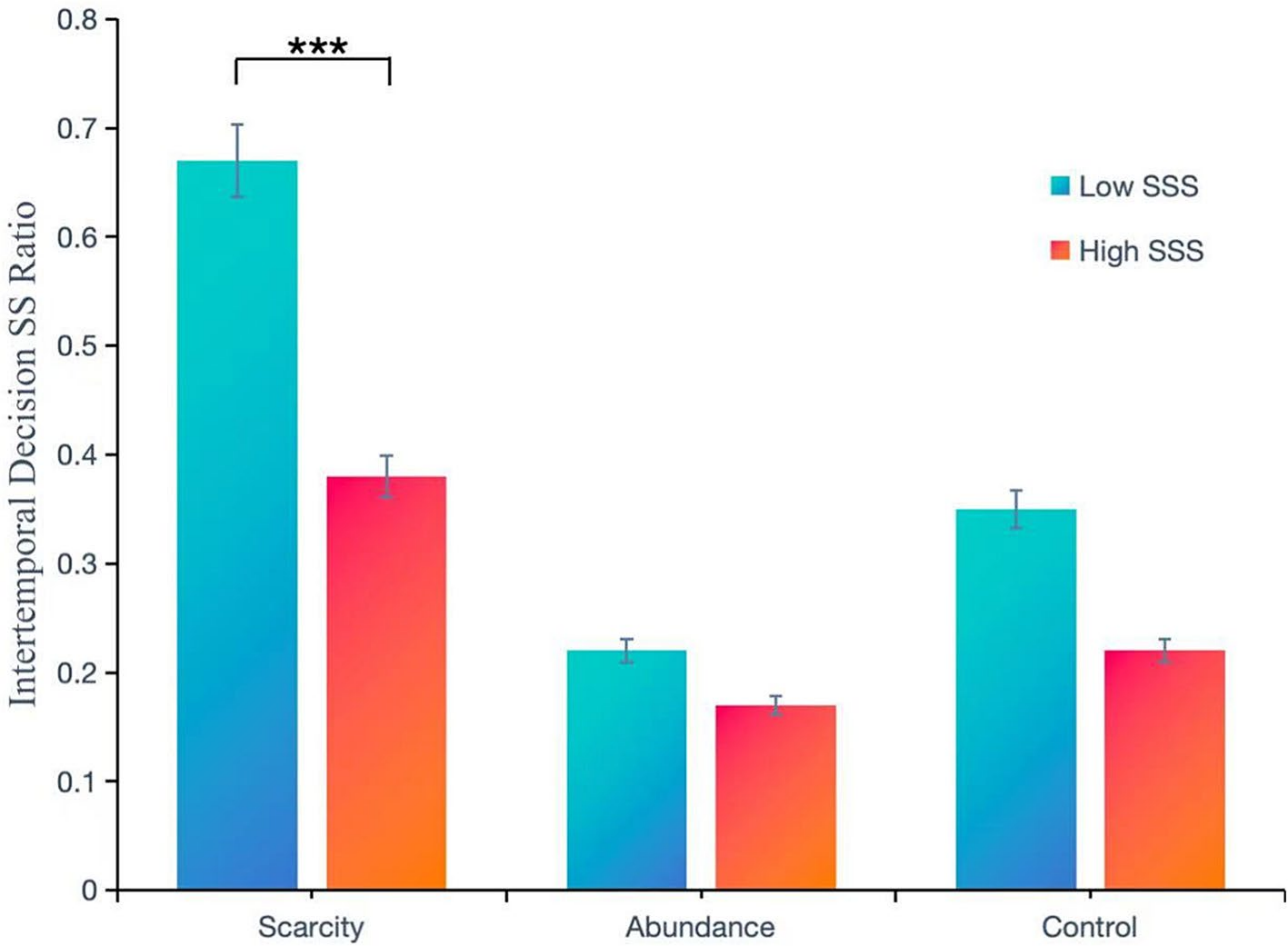
Differences in the SS ratios of individuals with varying subjective socioeconomic statuses in intertemporal decision-making under resource scarcity, ***p < 0.001.

In the resource scarcity group, the SS ratios of intertemporal decisions differed significantly between low SSS and high SSS, *F*(1,292) = 20.61, *p* < 0.001, *η*^2^ = 0.07. The SS ratio for the low SSS group (*M* ± SD = 0.67 ± 0.30) was significantly higher than that of the high SSS group (*M* ± SD = 0.38 ± 0.37). For the resource-rich and control groups, there was no significant difference in SS ratios between the low SSS and high SSS groups, *p* > 0.05 (Figure 2).

### 2.4 Discussion

Experiment 1 utilized a choice task within the framework of intertemporal decision-making to investigate whether individuals with different subjective socioeconomic statuses exhibit differences in their decision-making preferences under conditions of resource scarcity, non-scarcity, and resource abundance. The measurement of subjective socioeconomic status was conducted using the Chinese versions of the Childhood and Adulthood Subjective Socioeconomic Status Scales (Griskevicius et al., 2011).

Resource scarcity was induced using reading tasks (Griskevicius et al., 2013) and writing tasks (Hill et al., 2016), with the reading materials adapted based on the specific social context and characteristics of the participant group. The results of Experiment 1 were consistent with the hypothesis, demonstrating that both resource scarcity and subjective socioeconomic status significantly influenced individuals’ intertemporal choice preferences. Specifically, individuals with low subjective socioeconomic status exhibited a stronger preference for immediate rewards compared to those with high subjective socioeconomic status. In the resource scarcity group, participants showed a higher rate of immediate choices than those in the control and resource-rich groups. This aligns with previous research indicating that youth from impoverished backgrounds are more pessimistic about the future than their peers from affluent communities (Ramos et al., 2013). Moreover, less affluent students tend to choose quicker, less resource-intensive careers compared to their wealthier counterparts (Mishra et al., 2014).

Additionally, a significant interaction was found between resource scarcity and subjective socioeconomic status. In the resource scarcity group, both high and low subjective socioeconomic status individuals displayed a greater tendency to choose immediate rewards, illustrating that individuals respond adaptively to resource scarcity. Notably, significant differences in decision-making preferences between high and low subjective socioeconomic status individuals emerged only in the resource scarcity condition, suggesting that those with low subjective socioeconomic status are more sensitive to cues related to resource availability. Economic downturn cues appear to prompt individuals with lower childhood subjective socioeconomic status to prefer immediate rewards (Griskevicius et al., 2013). This further supports the view of differential susceptibility theory, indicating that individuals with low subjective socioeconomic status exhibit heightened sensitivity to threat cues in their environment, and their decision-making patterns are more readily shaped by external circumstances.

As mentioned earlier, individuals from lower subjective socioeconomic status backgrounds are more likely to be chronically exposed to resource scarcity and, consequently, tend to be more vigilant, act impulsively, and have a less optimistic outlook on the future compared to their higher-status counterparts (Griskevicius et al., 2013). Therefore, in resource scarcity tasks, individuals who perceive ecological cues of scarcity recognize the harshness of their environment, experience a reduced sense of control, and shift their time preference from the future to the present, capturing immediate resources as a reflection of adaptive behavior in response to their current circumstances.

The environment is essential for individual development, and life history strategies suggest that resource scarcity and environmental uncertainty serve as crucial cues for individuals to acquire experiences and develop behavioral patterns (Schaller et al., 2010). Thus, Experiment 1 examined the impact of resource scarcity as a threat cue on individuals with differing subjective socioeconomic statuses, while Experiment 2 will explore the effects of environmental uncertainty on choice preferences among individuals with varying subjective socioeconomic statuses.

## 3 Experiment 2: the impact of subjective socioeconomic status and environmental uncertainty on intertemporal decision making

### 3.1 Objective and hypotheses

Experiment 2 aims to explore the preferences in intertemporal decision-making among individuals with different subjective socioeconomic status under conditions of environmental uncertainty. The research hypotheses are as follows: H1: There is a significant main effect of subjective socioeconomic status. Individuals with low subjective socioeconomic status are more likely to choose the immediate option in intertemporal decision-making compared to those with high subjective socioeconomic status. H2: There is a significant main effect of subjective socioeconomic status. Individuals in the environmental uncertainty group are more likely to choose the immediate option in intertemporal decision-making compared to those in the control group and the environmental certainty group. H3: There is an interaction effect between subjective socioeconomic status and environmental uncertainty. In the environmental uncertainty group, individuals with low subjective socioeconomic status show a greater preference for the immediate option compared to those with high subjective socioeconomic status.

### 3.2 Methods

#### 3.2.1 Participants

Using G*Power 3.1 software, 2 × 3 ANOVA was conducted with *α* set at 0.05 and 1-*β* at 0.8, with an *effect* size of 0.25, resulting in a required sample size of 158 participants. The participants were employees from service-oriented technical fields and did not have a background in psychology. A total of 289 eligible participants were recruited, aged 18 to 65 years (*M* ± SD = 33.70 ± 9.61 years). They were randomly assigned to one of three groups: environmental uncertainty, control, and environmental certainty. The environmental uncertainty group consisted of 101 participants (32 in the high subjective socioeconomic status group and 69 in the low subjective socioeconomic status group, with 51 males and 50 females, *M* ± *SD* = 34.06 ± 9.89 years). The control group included 93 participants (31 in the high subjective socioeconomic status group and 62 in the low subjective socioeconomic status group, with 42 males and 51 females, *M* ± *SD* = 32.54 ± 8.88 years). The environmental certainty group had 95 participants (35 in the high subjective socioeconomic status group and 60 in the low subjective socioeconomic status group, with 44 males and 51 females, *M* ± *SD* = 34.46 ± 10.00 years). Participants were required to have basic reading and comprehension abilities and had not previously participated in similar experimental research. This study was approved by the Ethics Committee of the Psychology Department at Hunan Normal University (Approval Number: 2025-527).

#### 3.2.2 Experimental design

This experiment employs a 2 (Subjective Socioeconomic Status: High vs. Low) × 3 (Environmental Uncertainty: Uncertain vs. Control vs. Certain) between-subjects design. The independent variables are subjective socioeconomic status and environmental uncertainty. Subjective socioeconomic status is categorized as high or low based on the median score, where a higher score indicates a higher subjective socioeconomic status. The dependent variable is the proportion of participants choosing the immediate option.

#### 3.2.3 Measures

##### 3.2.3.1 Questionnaire on subjective socioeconomic status across life stages

Same as Experiment 1.

##### 3.2.3.2 Assessment of environmental uncertainty materials

To verify the effectiveness of the environmental uncertainty manipulation, participants completed a brief manipulation-check questionnaire immediately after the reading-and-writing priming task and before the intertemporal choice task. The questionnaire consisted of four items assessing perceived unpredictability and uncertainty regarding one’s future environment. Participants rated their agreement on a 7-point Likert scale (1 = strongly disagree, 7 = strongly agree). The items were: (1) “I feel uncertain about what will happen in my future”; (2) “It is difficult for me to predict my future life circumstances”; (3) “The environment around me feels unstable and unpredictable”; and (4) “I feel that future outcomes are largely beyond my personal control.” Item scores were averaged to form an overall perceived environmental uncertainty index, with higher scores indicating greater perceived uncertainty.

To ensure the effectiveness of the environmental uncertainty inducement materials, this study classified the materials based on relevant literature. We collected three types of reading materials: (1) materials related to environmental uncertainty (e.g., global challenges posed by pandemics); (2) neutral control materials (e.g., memory characteristics of aquatic organisms); and (3) positive materials concerning environmental certainty (e.g., reports of economic recovery). Subsequently, a pilot study was conducted to evaluate differences among these materials in terms of inducing environmental uncertainty, participant familiarity, and stimulation levels. The results indicated that the three groups of materials effectively differentiated the required state of environmental uncertainty while showing no significant differences in terms of participant familiarity and stimulation levels (Table 7).

**Table 7:** Results of one-way ANOVA for the manipulation check of uncertainty environment.

Level of uncertainty	*n*	*M*	SD	*F*	*p*
Uncertain	5	6.00	1.05	13.50	<0.001
Certain	5	3.27	0.64		
Control	5	3.80	0.90		

##### 3.2.3.3 Intertemporal decision-making task

Same as Experiment 1.

#### 3.2.4 Experimental procedure

All participants completed the experiment in individual rooms using E-Prime 2.0 software (a psychological software tool developed in Pittsburgh, Pennsylvania, USA) to present stimuli and collect behavioral data. At the beginning of the experiment, participants were required to fill out a questionnaire assessing their subjective socioeconomic status (SSS) during childhood, adolescence, and adulthood. Following this, participants were randomly assigned to one of three groups: one group read materials designed to induce environmental uncertainty, the second group read materials related to everyday events, and the third group read materials that reinforced environmental certainty. Each group was tasked with completing relevant writing assignments. After completing these tasks, all participants filled out a questionnaire related to uncertainty to verify the activation effects. Subsequently, participants engaged in an intertemporal decision-making task to evaluate their decision-making behavior. During the analysis, the consistency of subjective socioeconomic status across different periods was first examined, and childhood SSS scores were ultimately selected as the independent variable for further analysis.

#### 3.2.5 Data analysis and statistics

Data analysis was performed using SPSS 23.0. Specifically, Pearson correlation analyses were conducted on subjective socioeconomic status ratings from childhood, adolescence, and adulthood. A one-way analysis of variance (ANOVA) with post-hoc comparisons (LSD method) was applied to the manipulation check scores. To test the core hypothesis regarding the effects of environmental uncertainty and subjective socioeconomic status on the intertemporal decision SS ratio, a two-way ANOVA followed by simple effect analyses was employed, with gender and age included as control variables. Consistent with Experiment 1, interaction effects are interpreted as evidence that environmental uncertainty functions as a contextual moderator of SSS-related intertemporal preferences (see Figure 2).

### 3.3 Results

#### 3.3.1 Consistency of subjective socioeconomic status across life stages

A Pearson correlation was conducted to analyze the consistency among the subjective socioeconomic status during childhood, adolescence, and adulthood. The results indicated a highly significant positive correlation between childhood SSS and adolescent SSS, *r* = 0.91, *p* < 0.001; childhood SSS and adult SSS also showed a highly significant positive correlation, *r* = 0.49, *p* < 0.001. Moreover, adolescent SSS and adult SSS were positively correlated with a highly significant result, *r* = 0.52, *p* < 0.001. All three periods of SSS (childhood, adolescence, and adulthood) demonstrated significant positive correlations, with all correlation coefficients exceeding 0.4, indicating a moderate level of positive correlation. This suggests a strong consistency among subjective socioeconomic statuses across childhood, adolescence, and adulthood (Table 8).

**Table 8:** Results of consistency analysis for subjective socioeconomic status scores across different periods.

Subjective socioeconomic status	*M*	SD	Childhood SSS	Adolescence SSS	Adulthood SSS
Childhood SSS	3.65	1.60	1		
Adolescence SSS	3.68	1.54	0.90***	1	
Adulthood SSS	5.20	1.59	0.49***	0.52***	1

***p < 0.001.

#### 3.3.2 Manipulation check for environmental uncertainty priming

A one-way ANOVA was conducted with the level of uncertainty (Uncertain vs. Control vs. Certain) as the independent variable and the scores from the uncertainty check as the dependent variable. The results indicated significant differences in scores across the different environmental uncertainty conditions, *F*(2, 286) = 58.72, *p* < 0.001, necessitating further multiple comparisons using the Least Significant Difference (LSD) method. The results of the multiple comparisons revealed that the uncertainty group’s scores (*M* ± SD = 5.61 ± 1.13) were significantly higher than those of the control group (*M* ± SD = 4.58 ± 1.24), *p* < 0.001. Additionally, the control group’s scores were significantly higher than those of the certain group (*M* ± SD = 3.73 ± 1.27), *p* < 0.001, thereby confirming the effectiveness of the manipulation (Table 9).

**Table 9:** Results of the one-way ANOVA for the manipulation check of environmental uncertainty priming.

Level of uncertainty	*n*	*M*	SD	*F*	*p*
Uncertain	101	5.61	1.13	58.72	<0.001
Certain	95	3.73	1.27		
Control	93	4.58	1.24		

#### 3.3.3 Effects of environmental uncertainty and subjective socioeconomic status on intertemporal decision-making

A two-way fully randomized analysis of variance (ANOVA) was conducted, with uncertainty level (Uncertain vs. Certain vs. Control) and subjective socioeconomic status (low SSS vs. high SSS) as independent variables. Gender and age were included as control variables, while the intertemporal decision-making SS ratio served as the dependent variable (Table 10).

**Table 10:** Descriptive statistics of the intertemporal decision-making SS ratio under conditions of environmental uncertainty by subjective social status.

Variables in the study	Subjective socioeconomic status	Scarcity	Abundance	Control	Total
Intertemporal	Low SSS	0.67 ± 0.32	0.24 ± 0.25	0.36 ± 0.35	0.43 ± 0.35
Decision-making SS ratio	High SSS	0.43 ± 0.36	0.22 ± 0.18	0.30 ± 0.30	0.31 ± 0.30
(*M* ± SD)	Total	0.59 ± 0.35	0.23 ± 0.22	0.34 ± 0.34	0.39 ± 0.34

After controlling for the effects of gender and age, the main effect of Subjective Socioeconomic Status (SSS) was significant, *F* (1, 281) = 7.25, *p* < 0.01, *η*^2^ = 0.03. The intertemporal decision-making SS ratio for the low SSS group (*M* ± SD = 0.43 ± 0.35) was significantly higher than that of the high SSS group (*M* ± SD = 0.31 ± 0.30). The main effect of uncertainty was also significant, *F* (2, 281) = 25.12, *p* < 0.001, *η*^2^ = 0.15. Further *post hoc* comparisons revealed that the SS ratio for intertemporal decisions in the uncertainty group (*M* ± SD = 0.59 ± 0.35) was significantly higher than that of the control group (*M* ± SD = 0.34 ± 0.34), *p* < 0.001; additionally, the SS ratio in the control group was significantly higher than that in the certainty group (*M* ± SD = 0.23 ± 0.22), *p* < 0.05. There was a significant interaction between SSS and uncertainty, *F* (2, 281) = 3.06, *p* < 0.05, *η*^2^ = 0.02, necessitating a simple effects analysis (Table 11).

**Table 11:** Between-subjects effects of subjective social status and environmental uncertainty on intertemporal decision-making SS ratios.

Source of error	*SS*	*df*	*MS*	*F*	*p*	*η* ^2^
Intercept	2.13	1	2.13	22.87	0.01	0.08
Gender	0.06	1	0.06	0.61	0.43	0.00
Age	0.01	1	0.01	0.11	0.74	0.00
Resource group	4.67	2	2.34	25.12	0.01	0.15
SSS	0.67	1	0.67	7.25	0.01	0.03
Resource group * SSS	0.57	2	0.29	3.06	0.05	0.02
Error	26.13	281	0.09			
Total	78.85	289				

The results of the simple effects analysis indicate that, for individuals with low subjective socioeconomic status (SSS), there is a significant difference in the SS ratios of intertemporal decision-making across different uncertainty conditions, *F* (2, 281) = 34.11, *p* < 0.001, *η*^2^ = 0.20. Post-hoc multiple comparisons revealed that the SS ratio in the uncertainty group (*M* ± SD = 0.67 ± 0.32) was significantly higher than that in the control group (*M* ± SD = 0.36 ± 0.35), *p* < 0.001. Furthermore, the SS ratio in the control group was significantly higher than that in the certainty group (*M* ± SD = 0.24 ± 0.25), *p* < 0.05.

For individuals with high subjective socioeconomic status (SSS), significant differences in SS ratios of intertemporal decision-making were also observed across varying levels of certainty, *F* (2, 281) = 4.04, *p* < 0.05, *η*^2^ = 0.03. Post-hoc comparisons showed that the SS ratio in the uncertainty group (*M* ± SD = 0.43 ± 0.36) was significantly higher than that in the certainty group (*M* ± SD = 0.22 ± 0.18), *p* < 0.01. However, the SS ratio in the control group (*M* ± SD = 0.30 ± 0.30) did not significantly differ from either the uncertainty or certainty groups, *ps* > 0.05.

**Figure 3:**
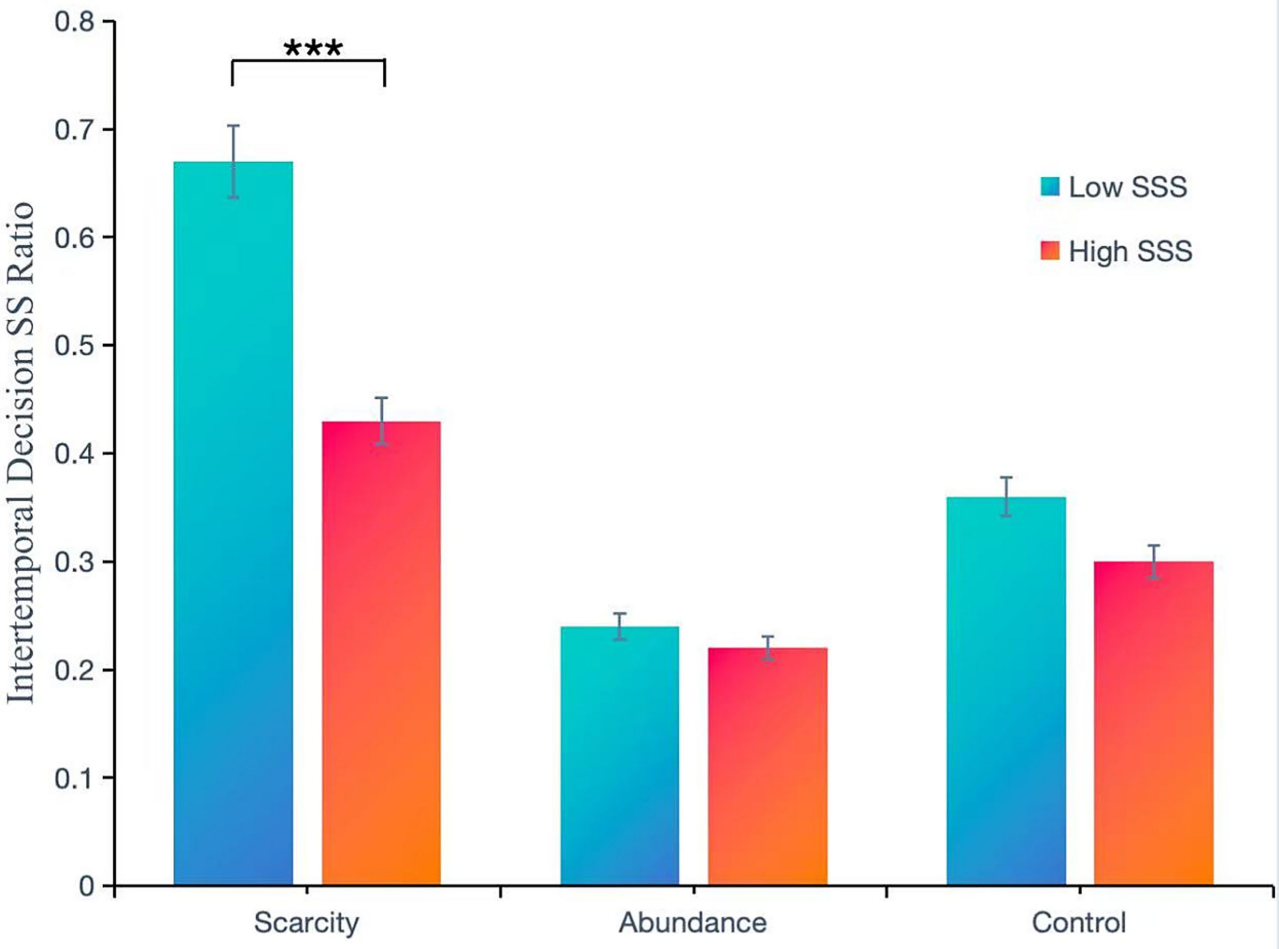
Differences in SS ratios of intertemporal decision-making across different subjective socioeconomic statuses under conditions of environmental uncertainty, ***p < 0.001.

In the uncertainty group, a significant difference was found in the SS ratios of intertemporal decision-making between individuals with low subjective socioeconomic status and those with high subjective socioeconomic status, *F* (1, 281) = 12.35, *p* < 0.01, *η*^2^ = 0.04. The SS ratio for the low SSS group (*M* ± SD = 0.67 ± 0.32) was significantly higher than that for the high SSS group (*M* ± SD = 0.43 ± 0.36). In contrast, for the certainty and control groups, there were no significant differences in the SS ratios of intertemporal decision-making between the low and high subjective socioeconomic status groups, *p* > 0.05(Table 12; Figure 3).

**Table 12:** Pairwise comparisons of the SS ratios in intertemporal decision-making under the environmental uncertainty groups.

(I)Uncertainty grouping	(J)Uncertainty grouping	Mean difference	95%CI	
			LLCI	ULCI
Uncertain	Certain	0.32*	0.23	0.41
	Control	0.22*	0.13	0.31
Certain	Uncertain	−0.32*	−0.41	−0.23
	Control	−0.10*	−0.19	−0.01
Control	Uncertain	−0.22*	−0.31	−0.13
	Control	0.10*	0.01	0.19

*p < 0.05.

### 3.4 Discussion

The measurement of subjective socioeconomic status and the intertemporal decision-making tasks in Experiment 2 were consistent with those in Experiment 1. The writing task under environmental uncertainty required participants to “draw from their own experiences,” aiming to examine whether there are differences in intertemporal decision preferences among individuals with different subjective socioeconomic status under conditions of environmental certainty, uncertainty, and determinacy. The results of Experiment 2 aligned with our hypotheses. There were no significant differences in subjective socioeconomic status across different periods. A significant main effect of environmental uncertainty was observed: under conditions of environmental uncertainty, the proportion of immediate choices made by individuals was significantly higher than that of the control group, while the control group significantly favored immediate choices over the environmentally definite group. This indicates that cues of environmental uncertainty increase individuals’ feelings of uncertainty, leading them to prefer immediate rewards.

The main effect of subjective socioeconomic status was significant, with individuals of lower subjective socioeconomic status showing a stronger preference for immediate options, which is consistent with previous research. This suggests that both environmental uncertainty and differing levels of subjective socioeconomic status influence individuals’ intertemporal choice preferences. Some studies have discovered that the uncertainty brought about by economic downturns leads those with lower socioeconomic status to focus more on the present, exhibit risk aversion, and engage in prosocial behaviors. It has also been shown to be associated with increased levels of obesity (Hill et al., 2016). If a person is frequently exposed to cues that make it impossible to predict income a month or even a year into the future, it is sensible for them to concentrate their efforts on meeting current needs rather than wasting energy on an uncertain future (Mittal et al., 2020).

Experiment 2 also revealed a significant interaction between socioeconomic status and environmental uncertainty. The difference in immediate choice rates between high and low subjective socioeconomic status individuals was significant only in the environmental uncertainty group, suggesting that individuals with low subjective socioeconomic status are more sensitive to cues of environmental uncertainty, Conversely, in the control group and the environmentally definite group, there were no significant differences in intertemporal choice preferences among individuals of varying subjective socioeconomic statuses, indicating that the prompt of environmental uncertainty can influence individuals’ choice preferences in a short time frame. This finding aligns with the hypothesis of Experiment 2.

Environmental uncertainty is considered a key aspect of living in low-income environments (Mittal and Griskevicius, 2016). socioeconomic status encompasses individuals’ perceptions of their economic standing. In an unequal society, lower and minimum wage earners may feel increasingly uncertain when compared to the stability of high-income earners regarding work schedules and income (Reyes and Yujuico, 2014). Therefore, environmental uncertainty brings about psychological effects of uncertainty (Geng et al., 2022), which can influence individuals’ choice preferences. For low subjective socioeconomic status individuals who frequently encounter uncertainty cues, sensitivity to environmental uncertainty prompts a greater tendency to opt for immediate rewards. However, when environmental information shifts towards a more positive context, the differences in intertemporal choice preferences across different levels of subjective socioeconomic status diminish.

## 4 General discussion

Our findings reveal that threat cues selectively potentiate the link between subjective socioeconomic status (SSS) and intertemporal decision-making. Clear divergences between high- and low-SSS individuals emerged specifically under conditions of resource scarcity and environmental uncertainty. In contrast, within resource-abundant and environmentally certain contexts, decision preferences converged across SSS levels, with a general tendency toward delayed gratification.

The core contribution of this study lies in moving beyond a simple verification of main effects. By integrating Differential Susceptibility Theory and Life History Theory, it systematically uncovers a theoretically grounded interactive mechanism between subjective socioeconomic status and different types of threat cues (resource scarcity vs. environmental uncertainty). The following discussion will delve into these findings through the theme of “From Stable Adaptive Dispositions to Context-Specific Activation.”

### 4.1 Baseline difference: the stable influence of subjective socioeconomic status on intertemporal decision-making

This study first validated a significant main effect of subjective socioeconomic status on intertemporal decision-making in a Chinese sample, specifically that individuals with low subjective socioeconomic status exhibit a stronger preference for immediate rewards. Although this finding aligns with the results of numerous Western studies, such as those by Pinger et al. (2024), Peviani et al. (2024), and Tunney and James (2022), its replication within the specific sociocultural context of China not only reinforces the universality of this effect but also establishes a solid baseline for the subsequent investigation of the moderating role of threat cues in this study.

In the present study, subjective socioeconomic status (SSS) was operationalized as individuals’ perceived social standing and was measured retrospectively across different life stages. The correlational analyses indicated that SSS ratings across childhood, adolescence, and adulthood were positively associated within this sample, suggesting a degree of consistency in perceived status across time points as assessed in a single measurement occasion. Importantly, however, the current design is cross-sectional and does not include longitudinal follow-up or test–retest assessments. Therefore, the present findings should not be interpreted as direct evidence that SSS is a long-term stable psychological construct. Future longitudinal research is needed to determine the temporal stability of SSS and to examine how within-person changes in perceived status relate to intertemporal decision-making over time. Existing research indicates that growing up in threatening environments significantly impacts individuals’ perceptions of socioeconomic status in adolescence and adulthood (Bean et al., 2024). For instance, prolonged exposure to environments marked by violence, discrimination, or economic stress may lead individuals to develop a deep-seated perception of social injustice, thereby influencing their career choices, educational investments, and interpersonal relationships. Furthermore, low socioeconomic status is closely associated with lower educational attainment and less stable career aspirations (Baker et al., 2014; Gutman and Schoon, 2012), which further corroborates the link between low subjective socioeconomic status and short-term oriented behaviors (Pepper and Nettle, 2017; Hill et al., 2016).

From the perspective of intertemporal decision-making, individuals with low subjective socioeconomic status often exhibit short-term decision tendencies, such as poor diabetes management, poorer physical health, and lower quality of life (Cerutti et al., 2024). These manifestations are closely linked to their experiences of low socioeconomic status, reflecting their future planning and self-evaluations of social standing (Liu et al., 2025). Under the influence of threat cues, individuals with low subjective socioeconomic status demonstrate a stronger preference for immediate choices in intertemporal decision-making, whereas in non-threatening environments, both high- and low-status individuals tend to favor delayed gratification. These findings robustly support the influence of subjective socioeconomic status on intertemporal decision preferences and the moderating role played by threat cues. Research has noted that individuals are highly sensitive to their position within the social hierarchy and adjust their cognitive and decision-making processes according to their social environment (Lee and Yoon, 2022). Individuals with low subjective socioeconomic status typically exhibit higher discounting of future benefits, which is closely related to their developmental environments and psychological perceptions (Joshi and Fast, 2013). For example, long-term habituation to resource-scarce environments may lead individuals to adopt a “fast life history strategy”, prioritizing immediate survival and reproduction over long-term planning and development. Studies also find that childhood experiences play a crucial role in shaping responses to external environmental stressors in adulthood (Griskevicius et al., 2011; Hill et al., 2012). In the absence of threats, decision-making may converge, whereas under threatening conditions, distinct decision preferences can emerge significantly. Therefore, understanding individuals’ decision preferences under threat requires considering the interactive influence of subjective socioeconomic status and environmental factors.

In summary, the influence of subjective socioeconomic status on intertemporal decision-making exhibits complex adaptive characteristics. The short-term choice preference displayed by individuals with low socioeconomic status under threat cues constitutes an adaptive behavior in response to resource scarcity and environmental uncertainty. Future research should further explore how to enhance the decision-making capacity of individuals with low subjective socioeconomic status by improving environmental factors, such as providing greater access to education and employment opportunities, strengthening social security systems, and enhancing social support networks, to promote their long-term healthy development. Additionally, psychological intervention methods could be investigated, such as boosting individuals’ sense of control, enhancing future orientation, and cultivating the ability to delay gratification, to help them overcome the pitfalls of short-termism and make wiser decisions.

### 4.2 Contextual activation: the moderating role of threat cues in intertemporal decision-making

Building on the baseline association between SSS and intertemporal decision-making, the present research further examined whether threat cues shape when and for whom SSS-related differences are most likely to emerge. Specifically, resource scarcity and environmental uncertainty were manipulated as situational signals of harshness and unpredictability. This allows us to move beyond a main-effect account and to test a context-activation perspective in which threat cues amplify short-term oriented preferences, particularly among individuals who perceive themselves as lower in socioeconomic standing.

Experimental results indicated that under threatening conditions of resource scarcity and environmental uncertainty, individuals exhibited a stronger preference for immediate rewards, while in environments characterized by resource abundance and certainty, their preferences shifted toward delayed gratification. This finding aligns with research by Sheehy-Skeffington and Jessica (2019), suggesting that individuals adapt their decision-making orientations in response to different environmental contexts when addressing life challenges and social circumstances. Throughout cycles of famine and abundance in human society, individuals in resource-scarce and uncertain environments often focus their decisions on short-term fulfillment. Evolutionary developmental theory posits that environmental characteristics during an individual’s growth process shape their cognitive processing and decision-making approaches (Griskevicius et al., 2011; Sheehy-Skeffington, 2019).

In the experiments, individuals in the resource scarcity group demonstrated a significantly higher preference for immediate choices, a decision-making process driven by the current state of resource shortage (Yang and Zhang, 2022; Zhang et al., 2023; Zeng et al., 2024; Yu et al., 2024). Studies have shown that under resource-scarce conditions, individuals face limited cognitive resources and heightened anxiety, leading them to prioritize addressing immediate and pressing needs (Petersohn et al., 2024). For instance, when individuals face threats to basic needs such as food, housing, or healthcare, they may be more inclined to choose options that satisfy these needs immediately, often overlooking long-term benefits. Therefore, individuals in resource-deprived environments exhibit a stronger preference for immediate gratification, closely linked to the psychological processes triggered by threat perception.

In the environmental uncertainty group, individuals similarly displayed a marked preference for immediate rewards, primarily because environmental uncertainty depletes their self-control resources (Xia et al., 2017). In such contexts, heightened anxiety and stress lead individuals to favor short-term gains over long-term benefits. Construal level theory suggests that when confronted with imminent threats, individuals’ thinking patterns rapidly shift to proximal and concrete processing, whereas in stable environments, they tend to adopt more distal and abstract thinking (Mishra et al., 2014). For example, when facing uncertainties such as unemployment, illness, or social instability, individuals may be more inclined to choose options that provide immediate security and satisfaction, neglecting long-term planning and development.

However, it is noteworthy that the influence of threat cues on intertemporal decision-making is not always negative. In some cases, threat cues may also prompt individuals to adopt more cautious and rational decisions. For instance, when facing a severe financial crisis, individuals may be more likely to seek professional financial advice, develop detailed budget plans, and take proactive steps to improve their financial situation. Thus, the impact of threat cues on intertemporal decision-making is complex and moderated by various factors, such as an individual’s cognitive ability, emotion regulation skills, and social support networks.

In summary, threat cues profoundly influence intertemporal decision-making by affecting individuals’ cognitive resources and psychological states. Under conditions of resource scarcity and environmental uncertainty, individuals exhibit a clear trend toward short-termism in their decision-making, whereas in benign environments, they lean more toward delayed gratification. This study provides empirical support for understanding shifts in intertemporal decision preferences across different environments and lays a foundation for future interventions targeting decision-making among individuals of varying socioeconomic statuses. Future research could further explore the effects of different types of threat cues on intertemporal decision-making and examine individual differences in sensitivity to such cues.

### 4.3 Interaction effect: the joint influence of threat cues and subjective socioeconomic status on intertemporal decision-making

The core innovation of this study lies in its systematic revelation of significant and theoretically profound interactions between subjective socioeconomic status (SSS) and different types of threat cues (resource scarcity and environmental uncertainty). This finding goes beyond simplistic, intuitive assumptions such as “the disadvantaged become more disadvantaged” or “adding insult to injury.” Instead, grounded in Differential Susceptibility Theory and Life History Theory, it provides an in-depth explanation of the inherent sensitivity of individuals with low SSS to specific threat cues and the dynamic activation mechanism of their decision-making patterns.

Specifically, individuals with low subjective socioeconomic status exhibited a stronger preference for immediate options compared to those with high subjective socioeconomic status under conditions of resource scarcity and environmental uncertainty. This heightened sensitivity among low-SSS individuals can be theoretically explained by integrating Life History Theory with the Biological Sensitivity to Context model. Long-term exposure to environmental harshness and unpredictability may sensitize individuals’ cognitive and motivational systems, making them more reactive to situational cues signaling potential instability. In contrast, individuals with high subjective socioeconomic status are more likely to possess psychological buffer mechanisms—including stronger perceived control, greater future certainty, and more stable self-regulatory capacity—which dampen the impact of threat cues on their decision-making processes. As a result, threat cues function as contextual activators that selectively trigger fast life history strategies among low-SSS individuals, while exerting weaker effects on high-SSS individuals. In contrast, no significant difference in intertemporal decision preferences was observed among individuals under conditions of resource abundance and environmental certainty. This indicates that individual decision-making is the product of an interaction between socioeconomic factors and psychological resources.

In relatively affluent societies, perceptions of social inequality can heighten individual’ sensitivity to their own social status (Williams et al., 2019). Individuals with lower subjective socioeconomic status often demonstrate more pronounced discounting of the future, influenced not only by their economic circumstances but also by the interplay between their self-perception and the social environment (Mittal et al., 2020). For example, long-term experiences at the lower strata of society may expose individuals to greater discrimination and exclusion, along with a lack of social resources and opportunities, which can negatively impact their sense of self-worth and social standing. This negative impact may further reinforce their short-term orientation, making them more inclined to choose options that provide immediate gratification.

Life History Theory posits that individuals’ adaptive strategies are shaped by their developmental environments. Those in resource-scarce environments are more likely to adopt a “fast” strategy, whereas those in resource-abundant environments tend toward a “slow” strategy emphasizing delayed rewards (Mishra et al., 2014; Ellis et al., 2009). A fast life history strategy emphasizes rapid reproduction, early maturation, and quick adaptation to the immediate environment, while a slow strategy emphasizes delayed reproduction, later maturation, and long-term planning for the future. Due to prolonged exposure to resource-scarce and uncertain environments, individuals with low subjective socioeconomic status may lean more toward a fast life history strategy, thereby exhibiting a stronger preference for immediate choices.

Research shows that adults who experienced different childhood environments may behave similarly under non-threatening conditions but display markedly different decision-making styles when facing adversity and threats (Griskevicius et al., 2011). For instance, individuals who grew up in impoverished households may experience heightened anxiety and panic when confronted with economic stress, leading to more myopic decisions. Therefore, understanding individual decision preferences requires considering the role of both external environmental and internal socioeconomic factors in the decision-making process. These findings underscore that when designing individual interventions, reducing perceived threat and uncertainty in the environment can bolster the self-confidence of individuals with low subjective socioeconomic status, thereby enhancing their capacity for delayed gratification.

Recent studies also suggest that low socioeconomic status may, in some contexts, positively influence judgment and decision-making (Brink and Costigan, 2023). For example, underestimating task completion time may lead to less planning fallacy and reduced stress (Pezzo et al., 2006; Shmueli et al., 2016), while also providing certain adaptive advantages in the face of threats. Thus, there is no absolute superiority or inferiority in the decision-making of individuals from different socioeconomic backgrounds; rather, it reflects an adaptation to their current environment, further emphasizing the need to understand individual decisions within specific contextual conditions.

In summary, this study confirms that threat cues moderate the influence of subjective socioeconomic status on intertemporal decision preferences. Furthermore, exploring the multiple dimensions of the relationship between poverty and short-sightedness provides new perspectives for psychological theory. Considering individual cognition and behavior within an adaptive theoretical framework aids in developing more effective interventions to promote the long-term development of individuals across different socioeconomic statuses. Future research could further investigate the impact of different types of interventions—such as cognitive behavioral therapy, social skills training, and economic empowerment programs—on the intertemporal decision-making of individuals with low subjective socioeconomic status. Additionally, exploring the influence of cultural factors on intertemporal decision-making and how cultural resources can be leveraged to foster long-term individual development presents a promising avenue for further inquiry.

### 4.4 Limitations and directions for future research

While this study reveals the significant impact of subjective socioeconomic status and threat cues on intertemporal decision-making, further investigation into the underlying psychological mechanisms is necessary. Factors such as perceived control, anxiety levels, or future time perspective, for instance, warrant deeper exploration to understand their roles in this process. We have identified “what” occurs and “under what conditions” individuals with low subjective socioeconomic status exhibit a stronger preference for immediate choices. However, the internal psychological processes—specifically “how” and “why” these phenomena occur—remain a “black box.” For example, although we observed that individuals with low subjective socioeconomic status are more inclined to choose immediate rewards under threat cues, we have yet to delve into the psychological mechanisms behind this decision preference, such as how perceived control, anxiety levels, or future time perspective function in this process.

Secondly, discussions on ecological validity remain insufficient. There may be significant differences between laboratory-induced threats and the intensity or duration of real-life threats. In laboratory settings, the presentation of threat cues and individual responses may differ substantially from real-world scenarios. For instance, the materials used to simulate resource scarcity and environmental uncertainty in experiments may not fully replicate the complex situations individuals face in actual life. Therefore, future research should consider ways to enhance ecological validity to better reflect decision-making processes in real-life contexts.

Finally, the diversity and representativeness of the sample also represent a limitation that requires attention. Although this study included individuals across different age groups, the sample was primarily drawn from specific socioeconomic backgrounds, which may limit the generalizability of the findings. Future research should consider broader and more diverse samples to improve the external validity of the results.

## 5 Conclusion

This study explored the effects of subjective socioeconomic status and threat cues (resource scarcity and environmental uncertainty) on individuals’ intertemporal decision-making through two experiments. The findings can be summarized as follows: Firstly, subjective socioeconomic status significantly influences intertemporal decision-making, where individuals with lower subjective socioeconomic status show a greater preference for immediate rewards compared to those with higher subjective socioeconomic status. Secondly, resource scarcity significantly affects intertemporal decision-making; the resource scarcity group displayed a stronger preference for immediate rewards than both the control group and the resource-rich group. Additionally, environmental uncertainty also impacts intertemporal decision-making, with the environmental uncertainty group demonstrating a higher preference for immediate rewards compared to both the control group and the environmental certainty group.

Further analysis revealed that resource scarcity moderates the relationship between subjective socioeconomic status and intertemporal decision-making. In the resource scarcity group, individuals with lower subjective socioeconomic status showed a significantly greater preference for immediate rewards than those with higher subjective socioeconomic status. In contrast, there were no significant differences in intertemporal decision-making choices between individuals of differing subjective socioeconomic status in the control and resource-rich groups. Similarly, environmental uncertainty also played a moderating role between subjective socioeconomic status and intertemporal decision-making; in the environmental uncertainty group, individuals with lower subjective socioeconomic status preferred immediate rewards markedly more than those with higher subjective socioeconomic status, while no significant differences were observed in the control and environmental certainty groups.

## Data Availability

The datasets presented in this study can be found in online repositories. The names of the repository/repositories and accession number(s) can be found in the article/supplementary material.
