# Correction: The impact of subjective socioeconomic status and threat cues on intertemporal decision-making

**DOI:** 10.3389/fpsyg.2026.1818793

**Published:** 2026-03-16

**Authors:** Zhen Zeng, Yufan Chen, Weiguo Qu

**Affiliations:** 1 Cognition and Human Behavior Key Laboratory of Hunan Province, School of Education Science, Hunan Normal University, Changsha, China; 2 School of Education Science, Huaihua University, Huaihua, Hunan, China; 3 Wulingshan K-12 Educational Research Center, Huaihua University, Huaihua, Hunan, China

**Keywords:** environmental uncertainty, intertemporal decision-making, resource scarcity, subjective socioeconomic status, threat cues

Author Zhen Zeng was erroneously assigned as corresponding author. The correct corresponding author is Weiguo Qu.

The original version of this article has been updated.

